# The perceived relevance, utility and retention of basic sciences in general practice

**DOI:** 10.1186/s12909-024-05750-2

**Published:** 2024-07-29

**Authors:** Faith O. Alele, Francis A. Albert, Emma Anderson, Abdul-Aziz Seidu, Hannah Mason, Paula Heggarty, Aaron Hollins, Tarun Sen Gupta, Lawrie McArthur, Richard B. Hays, Bunmi S. Malau-Aduli

**Affiliations:** 1 College of Medicine and Dentistry, James Cook University, Townsville, QLD 4811 Australia; 2 School of Health, University of the Sunshine Coast, Sunshine Coast, QLD 4556 Australia; 3 College of Public Health, Medical and Veterinary Sciences, James Cook University, Townsville, QLD 4811 Australia; 4 Discipline of General Practice, The University of Adelaide, Adelaide, SA 5005 Australia; 5 Adelaide Rural Clinical School, University of Adelaide, Adelaide, SA 5606 Australia; 6 School of Medicine and Public Health, College of Health, Medicine and Wellbeing, The University of Newcastle, Callaghan, NSW 2308 Australia

**Keywords:** Medical education, Basic sciences, Knowledge retention, Relevance, Postgraduate medical trainees, General practitioners

## Abstract

**Background:**

Basic sciences are crucial for clinical medicine, yet studies focusing on their perceived utility among general practitioners (GPs) are sparse. Considering the broad scope of GPs’ practice, an in-depth understanding of basic sciences is fundamental for making informed clinical decisions. This study evaluated GP registrars’ retention and perceptions of the utility of basic sciences in clinical practice.

**Methods:**

Using sequential explanatory mixed methods study design, knowledge retention was assessed by a multiple-choice question (MCQ) examination followed by interviews on the perception of the relevance and utility of basic sciences among GP registrars at James Cook University's (JCU) General Practice Training (GPT) program. Descriptive and inferential statistical analyses were conducted on the MCQ exam data, while thematic analysis was employed for the qualitative interview data.

**Results:**

Sixty-one GP registrars participated in the MCQ exam, while 11 of them were involved in the interviews. The highest mean score was obtained in biochemistry (75.1 ± 2.23) while the lowest mean score was in anatomy (56.07 ± 3.16). Key performance predictors included the formative clinical examination scores (β = 0.83, 95% CI: 0.45 to 1.2, *p* < 0.001) and gender (β = -9.7, 95% CI: -17 to -2.3, *p* = 0.011). The qualitative data analysis revealed five themes, including the backbone of clinical medicine, varying utility over time and by specialty, clinical synthesis integrates encapsulated knowledge, professional pressures hinder revisitation of knowledge and knowledge renewal enhances updates.

**Conclusion:**

Basic sciences were considered relevant in clinical practice. Development of continuing professional development (CPDs) sessions and clinically relevant online resources were measures proposed to enhance the retention of knowledge. Future research could focus on innovative educational strategies for GPs.

**Supplementary Information:**

The online version contains supplementary material available at 10.1186/s12909-024-05750-2.

## Introduction

Basic sciences are foundational to clinical medicine, significantly influencing medical education and practice [1, 2]. Hence, despite a shift towards competency-based medical education, the importance of basic sciences persists [3–5]. Nonetheless, research indicates a decline in basic science retention post-graduation, with medical graduates retaining 67% to 75% of their knowledge in the first year, decreasing to below 50% by the second year [6]. This challenge is also documented among undergraduate medical students, particularly during the clinical years [4–8]. Contributing factors include the limited coverage of basic sciences in clinical textbooks and the vast number of clinical facts that need to be memorised [4, 5].

Limited evidence among postgraduate medical trainees revealed poor knowledge retention rates, which was influenced by curriculum type, gender and age, suggesting that exposure to traditional curriculum, being female and shorter interval from graduation fostered basic science knowledge retention [9]. Despite low retention rates of basic sciences, medical practitioners often seek and apply this foundational knowledge when faced with complex medical cases [10, 11]. This knowledge is utilised in patient communication, diagnosis, and treatment selection, with its perceived clinical relevance contributing to retention [7].

Existing studies on the retention of basic sciences among doctors have primarily focused on interns and surgeons, overlooking general practitioners (GPs), who are crucial in primary healthcare delivery [12, 13]. GPs require a robust understanding of basic sciences to make informed clinical judgements, enhancing diagnostic precision and decision-making [1, 13, 14, 15]. Hence, it is imperative to understand GPs' level of basic sciences knowledge retention and their perceived role of basic sciences in clinical practice. These insights can guide the development of strategies to improve patient care and inform the utility of basic sciences in general practice.

Therefore, this study addressed the following research questions:

1. What is the relationship between basic sciences knowledge retention and cultural background, age and gender?
2. What is the relationship between GP registrars’ performance in basic sciences exams and other assessments?
3. What are GP registrars’ perceptions of the relevance and application of basic sciences knowledge into their clinical practice?

## Methods

### Study context

The study was conducted within the General Practice Training (GPT) program at James Cook University (JCU), which was established in 2016 [16]. Designed to serve regional, rural, and remote communities, the program focuses on integrated education in general practice and rural generalist medicine, spanning from undergraduate to clinician years. Trainees begin hospital rotations in their Post Graduate Years 1 and 2 and commence general practice or rural hospital training in at least their third postgraduate year. The first year of training spans over two semesters, followed by a second year focused on clinical practice and fellowship examinations with either the Australian College of Rural and Remote Medicine or the Royal Australian College of General Practitioners. JCU’s GPT program incorporates an internal assessment schedule, enabling the evaluation of trainees' academic progress from the onset of orientation. This formative assessment process involves educational diagnosis to identify trainees' learning requirements, facilitating early remediation plans, and supporting their successful completion of the training [17].

### Study design

Using a sequential explanatory mixed-methods approach [18], this study was conducted from June to December, 2023. The study commenced with the administration of a Basic Science Retention Examination (BSRE) which comprised 30 basic sciences MCQs to the registrars to assess their retention of basic sciences knowledge, in addition to the standard 65 MCQ clinical exam which is an integral component of JCU’s GPT program’s formative assessments. Insights from the quantitative data informed the development of the interview protocol and selection of participants for the qualitative phase. Subsequent individual interviews conducted via Microsoft Teams provided in-depth perspectives on the relevance and application of basic sciences in clinical practice. The integration of findings from both phases facilitated a comprehensive interpretation of the data, adhering to the Good Reporting of a Mixed-Methods Study (GRAMMS) standards [19]. The full checklist is included in Supplementary File 1.

### Target population

This study involved general practice residents, also known as registrars, enrolled in the JCU GPT program. These healthcare professionals, often the first point of contact for patients in regional, rural and remote communities, provide a broad range of medical services. The 2023 cohort of JCU GPT registrars commencing their training (GPT 1) and those who had commenced eight months prior (GPT 2) were invited in August 2023 to participate in the BSRE, in addition to their routine formative assessment. There was a total of 82 registrars in this cohort.

### Data collection

JCU GPT registrars were required to take a 65-question MCQ clinical examination in August 2023. For the quantitative part of this study, the BSRE, which comprised 30 basic sciences MCQs was administered to the registrars to assess their retention of basic sciences knowledge. The BSRE and clinical examination were combined into one, with a total of 95 MCQs and 110 minutes to complete the examination.

#### Quantitative data—test construction

All the 82 GPT 1 and GPT 2 registrars were invited to complete the combined BSRE (30 questions) and clinical (65 questions) examinations in August 2023. The registrars were informed 2 weeks prior about the duration of the examination, the online platform (testportal.net), the type of feedback that would be provided. Demographic variables of the participants were retrieved from the University Record System database. These variables include age, gender, years since graduation and graduate type—International Medical Graduates (IMGs) and Australian Medical Graduates (AMGs). Personalised feedback was offered as part of the routine formative assessments to enhance the registrars' engagement and their learning experience. The BSRE was designed to assess six core areas of basic sciences: anatomy, biochemistry, pathology, pharmacology, physiology, and social sciences, with five (5) questions per discipline. The basic sciences questions were sourced from the International Databases for Enhanced Assessments and Learning assessment item bank and were aligned with the basic science components of the curriculum [7, 20]. Each question presented a brief clinical scenario and required a single correct answer, and the examination was administered electronically. The validity of the test items was vetted by content experts. For comparison purposes, all examination scores were expressed as percentages of correct answers in both the overall test and individual disciplines.

#### Qualitative data

For the qualitative phase of the study, 46 registrars consented to be contacted for interviews, and 11 were available to participate. These participants were selected based on their availability, reflecting the demanding nature of their work schedules. Each participant received AUD 50 grocery voucher as an appreciation for their time. Individual interviews were conducted remotely by one of the investigators (FOA), using the call functionality of Microsoft Teams to explore perceptions of the clinical relevance and utility of basic sciences in general practice. All interviews were conducted over a single call, lasted between 25 to 40 minutes and were structured around eight predefined questions (see Supplementary File 2). The interviews were recorded with the participants’ consent and transcribed verbatim.

### Data analysis

Quantitative data analysis was conducted using R version 4.3.1 (Team, 2023). Categorical variables were presented as counts and percentages, while continuous variables were presented as medians and interquartile ranges (IQR). Comparative analyses employed Chi-square or Fisher’s exact tests for categorical variables and Kruskal–Wallis rank sum tests for continuous variables after checking for data normality. Pearson’s correlation test was conducted to assess the association between the different basic science disciplines, years since graduation, age, formative clinical exam score, and BSRE score. Multiple linear regression analysis was used to evaluate the influence of training background, age, and gender on the retention of basic science knowledge, while a multivariate linear model examined the factors affecting the observed score variations in the BSRE components.

Qualitative inductive thematic analysis [21] of the transcripts was conducted by three investigators (FOA, EA, and BSM-A), using NVivo 14. This involved the systematic identification of patterns, organisation into codes, and the development of themes [22], with discrepancies resolved through consensus meetings to ensure accuracy of the results. Integration of the quantitative and qualitative findings provided a comprehensive exploration of the results, enhancing the study’s depth and credibility.

### Ethical considerations

This study was conducted in accordance with the Declaration of Helsinki [23]. Participants were required to provide electronic consent for the MCQ examination and verbal consent for the interviews, ensuring research integrity. Ethics approval (H9140) for the study was granted by the James Cook University Human Research Ethics Committee (HREC). During recruitment and data collection, participants were fully informed about the ethical clearance, the study’s objectives, their privacy rights, and potential benefits, ensuring all participants were well-informed and their rights respected throughout the study.

## Results

### Quantitative findings

**Fig. 1:**
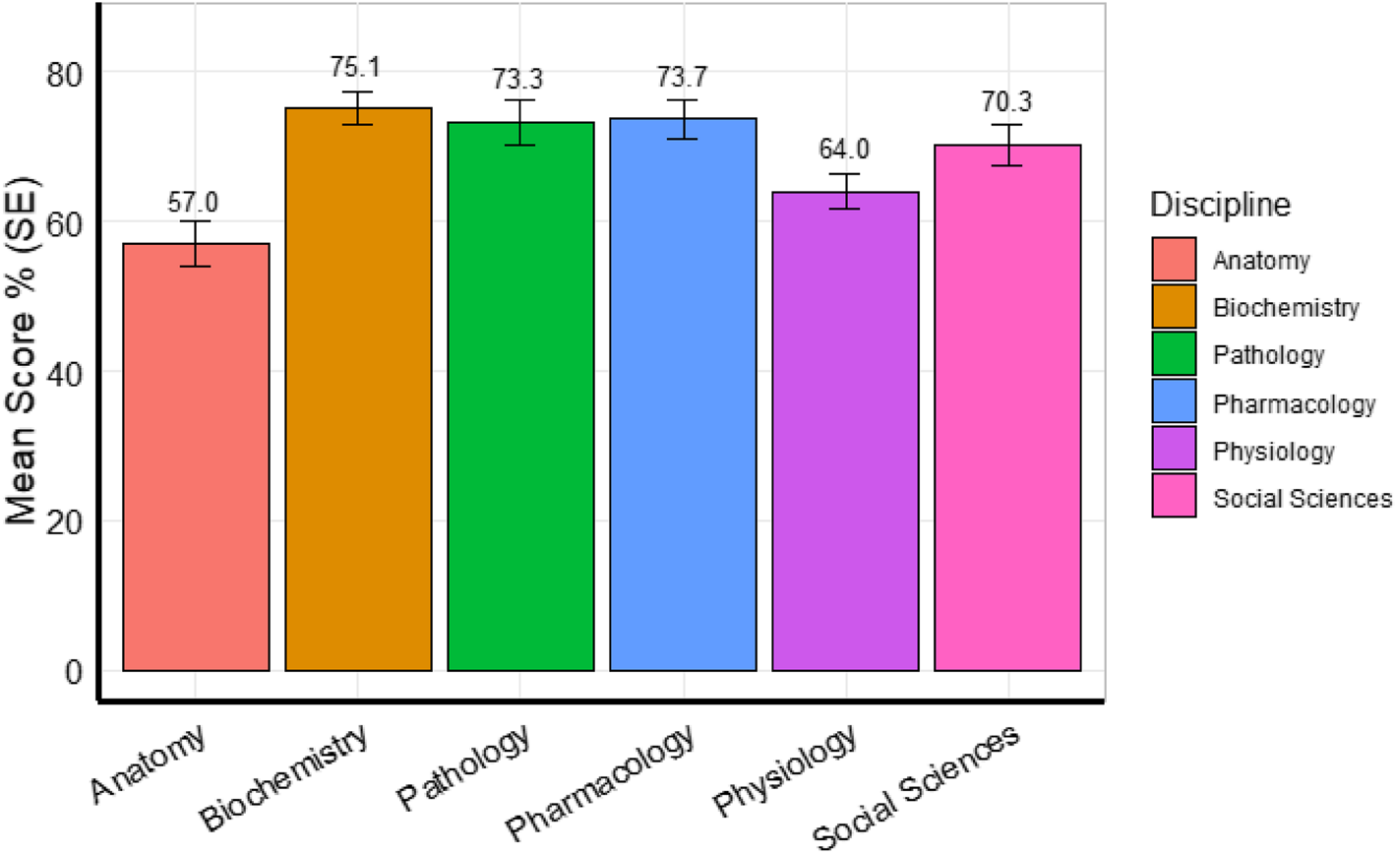
Mean scores for the basic science disciplines

A total of 61 registrars (74% response rate) participated in the quantitative phase of the study. The median age was 32 years (IQR: 29 – 38), with 72% females and 59% AMG. The median years post-graduation was 5 years. The range of percent scores for the GP formative clinical exam and the BSRE were 41.5—76.9 and 20 – 90, respectively, while the median scores were 62 (IQR: 54 – 66) and 70 (IQR: 57 – 77), respectively (Table 1). As shown in Fig. 1, scores varied across basic science disciplines, with the lowest in anatomy (56.07 ± 3.16), and the highest in biochemistry (75.08 ± 2.23).

**Table 1:** Study participants’ profile^a^

Variable	Numbers
**Age**, Median (IQR)	32 (29 – 38)
**Gender, n (%)**	
Female	44 (72)
Male	16 (26)
**Graduate type, n (%)**	
AMG	36 (59)
IMG	25 (41)
**Year Since Graduation**, Median (IQR)	5 (4 – 9)
**Formative clinical examination score**, Median (IQR)	62 (54 – 66)
**BSRE Score**, Median (IQR)	70 (57 – 77)

^a^Total number of participants (*N* = 61); noting one missing data for gender

**Fig. 2:**
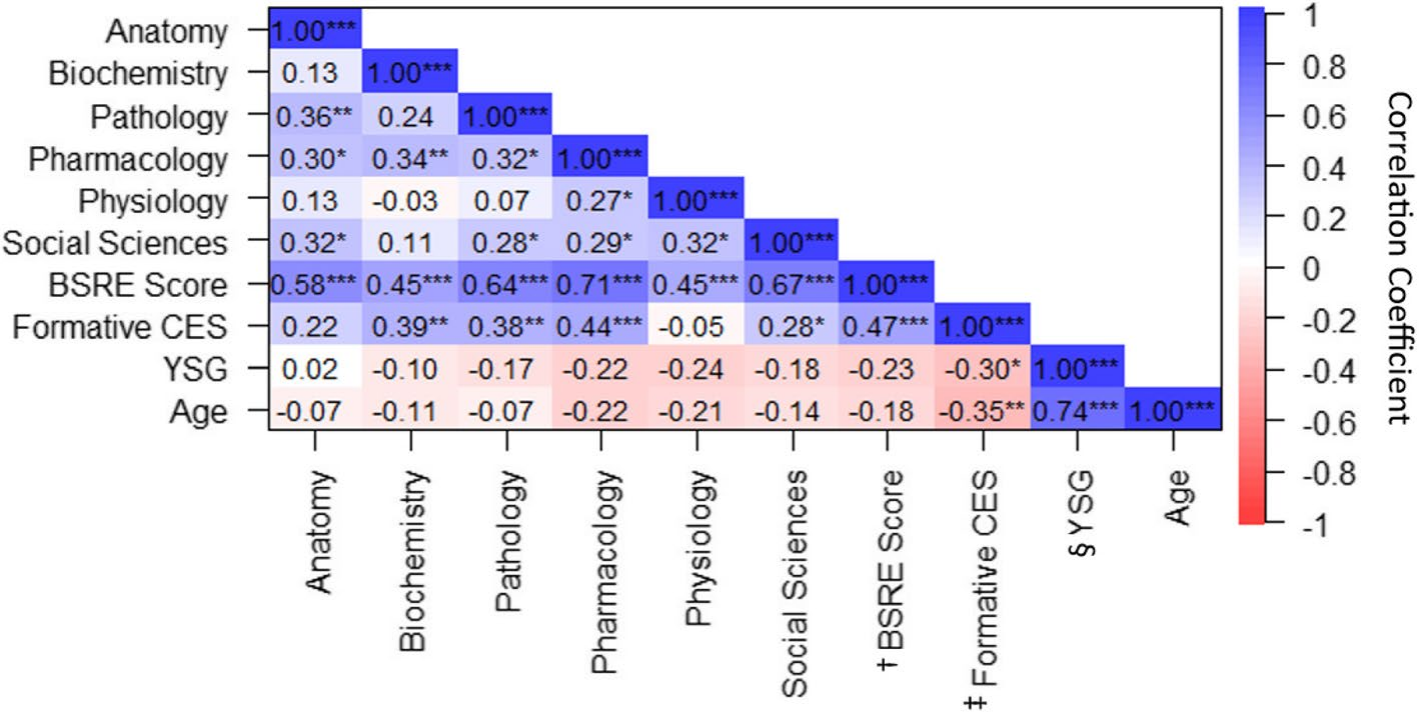
Associations between basic science disciplines, formative clinical examination score, years since graduation and age †Basic science retention examination score; ‡ Formative clinical examination score; §Years since graduation. *P < 0.05; **P < 0.001; ***P < 0.001

Figure 2 shows the correlation, between the basic science disciplines (anatomy, biochemistry, pathology, pharmacology, physiology, social sciences), years since graduation, age, formative clinical examination score and BSRE score. The correlation coefficients indicate a range of significant (*p* < 0.05) weak to strong associations. The overall BSRE score was positively and strongly correlated with pharmacology (*r* = 0.71, *p* < 0.001) and social sciences (*r* = 0.67, *p* < 0.001); it was moderately correlated with anatomy (*r* = 0.58, *p* < 0.001), biochemistry (*r* = 0.45, *p* < 0.001), pathology (*r* = 0.64, *p* < 0.001), physiology (*r* = 0.45, *p* < 0.001), and formative clinical examination (*r* = 0.47, *p* < 0.001). The formative clinical examination was positively correlated with biochemistry (*r* = 0.39, *p* < 0.01), pathology (*r* = 0.38, *p* < 0.01), social sciences (*r* = 0.28, *p* < 0.05) and pharmacology (*r* = 0.44, *p* < 0.001). Additionally, BSRE (not significant) and the formative clinical examination were negatively correlated with years since graduation (*r* = -0.30, *p* < 0.05) and age (*r* = -0.35, *p* < 0.01). Intercorrelations between the basic science disciplines showed that Pharmacology was positively correlated with the other basic science disciplines: anatomy (*r* = 0.30, *p* < 0.05), biochemistry (*r* = 0.34, *p* < 0.01), pathology (*r* = 0.32, *p* < 0.05), physiology (*r* = 0.27, *p* < 0.05), and social sciences (*r* = 0.29, *p* < 0.05). Social sciences was positively correlated with anatomy (*r* = 0.32, *p* < 0.05), pathology (*r* = 0.28, *p* < 0.05), pharmacology (*r* = 0.29, *p* < 0.05) and physiology (*r* = 0.32, *p* < 0.05).

The relationship between the overall BSRE score and the demographic variables was assessed using both univariate and multivariable regression analyses. As shown in Table 2, age, gender, and years since graduation were not significantly associated with the overall BSRE score in the univariate analysis. However, a statistically significant positive association was found between the formative clinical examination score and the overall BSRE score. Specifically, for every one-unit increase in the formative clinical examination score, there was an associated 0.79 units increase in the BSRE score (β = 0.79, 95% CI: 0.40—1.2, *p* < 0.001). The IMGs scored 9.3 units lower on the BSRE compared to the AMGs (β = -9.3, 95% CI: -16 to -2.3, *p* = 0.011). However, this association lost significance in the multivariable analysis (β = -8.8, 95% CI: -18 to 0.81, *p* = 0.072). The only significant predictors of the BSRE score in the multivariable analysis were the formative clinical examination (β = 0.83, 95% CI: 0.45 to 1.2, *p* < 0.001) and gender (β = -9.7, 95% CI: -17 to -2.3, *p* = 0.011). High performance in the formative clinical examination and being male were significantly and positively associated with high BSRE score.

**Table 2:** Predictors of performance in the basic sciences examination

	Univariate		Multivariable	
**Variable**	**Beta** **(95% CI)**1	***p*-value**	**Beta** **(95% CI)**1	***p*-value**
**Age**	-0.42 (-1.0 to 0.18)	0.165	-0.25 (-1.1 to 0.56)	0.529
**Gender**				
Male	Ref			
Female	-7.7 (-16 to 0.50)	0.065	-9.7 (-17 to -2.3)	**0.011**
**Formative clinical examination score**	0.79 (0.40 to 1.2)	** < 0.001**	0.83 (0.45 to 1.2)	** < 0.001**
**Years since graduation**	-0.55 (-1.2 to 0.07)	0.080	0.52 (-0.57 to 1.6)	0.342
**Graduate Type**				
AMG	Ref			
IMG	-9.3 (-16 to -2.3)	**0.011**	-8.8 (-18 to 0.81)	0.072

Table 3 presents the predictors of performance in each of the basic sciences disciplines. Gender was a significant predictor of performance in anatomy (β = -15, 95% CI: -31 to -1.8; *p* = 0.028), indicating anatomy score for females was 15 units lower compared to males. Similarly, IMGs’ scores in physiology (β = -17, 95% CI: -30 to -2.6, *p* = 0.021) and social sciences (β = -24, 95% CI: -40 to -7.9, *p* = 0.004) were 17 and 24 units lower (respectively) than AMGs' scores. However, a positive association was observed in their performance in biochemistry with 14 units higher than AMGs (β = 14; 95% CI; 0.35 – 27; *p* = 0.044). Formative clinical examination score showed a consistent and significant positive association with performance in biochemistry (β = 0.88; 95% CI; 0.34 to 1.4; *p* = 0.002), pathology (β = 1.2; 95% CI; 0.52 to 1.9; *p* = 0.001), pharmacology (β = 1.1; 95% CI 0.47 – 1.8; *p* = 0.001) and social sciences (β = 0.78; 95% CI; 0.08 – 1.4; *p* = 0.028).

**Table 3:** Predictors of performance in Anatomy, Biochemistry, Pathology, Pharmacology, Physiology and Social Sciences^a^

**Subjects**	**Characteristic**	**Beta** **(95% CI)**1	***p*-value**
Anatomy	Age	-0.79 (-2.4 to 0.81)	0.325
Anatomy	**Female vs Male**	**-15 (-31 to -1.8)**	**0.028**
Anatomy	Years since graduation	0.44 (-1.7 to 2.6)	0.679
Anatomy	Formative clinical examination score	0.73 (-0.05 to 1.5)	0.065
Anatomy	IMG vs AMG	7.4 (-12 to 27)	0.440
Biochemistry	Age	0.28 (-0.83 to 1.4)	0.618
Biochemistry	Female vs Male	-4.1 (-15 to 5.5)	0.358
Biochemistry	Years since graduation	-1.0 (-2.5 to 0.49)	0.185
Biochemistry	**Formative clinical examination score**	**0.88 (0.34 to 1.4)**	**0.002**
Biochemistry	**IMG vs AMG**	**14 (0.35 to 27)**	**0.044**
Pathology	Age	0.36 (-1.1 to 1.8)	0.617
Pathology	Female vs Male	-12 (-26 to 0.81)	0.065
Pathology	Years since graduation	0.23 (-1.7 to 2.2)	0.813
Pathology	**Formative clinical examination score**	**1.2 (0.52 to 1.9)**	**0.001**
Pathology	IMG vs AMG	-12 (-29 to 5.9)	0.189
Pharmacology	Age	-0.26 (-1.6 to 1.1)	0.694
Pharmacology	Female vs Male	-8.5 (-21 to 3.7)	0.170
Pharmacology	Years since graduation	-0.05 (-1.8 to 1.7)	0.958
Pharmacology	**Formative clinical examination score**	**1.1 (0.47 to 1.8)**	**0.001**
Pharmacology	IMG vs AMG	-1.6 (-18 to 1.7)	0.958
Physiology	Age	-0.70 (-1.8 to 0.45)	0.230
Physiology	Female vs Male	-2.3 (-13 to 8.3)	0.667
Physiology	Years since graduation	0.72 (-0.83 to 2.3)	0.354
Physiology	Formative clinical examination score	-0.25 (-0.82 to 0.32)	0.378
Physiology	**IMG vs AMG**	**-17 (-30 to -2.6)**	**0.021**
Social Sciences	Age	-0.67 (-2.0 to 0.65)	0.313
Social Sciences	Female vs Male	-10 (-22 to 1.9)	0.097
Social Sciences	Years since graduation	1.6 (-0.17 to 3.4)	0.075
Social Sciences	**Formative clinical examination score**	**0.73 (0.08 to 1.4)**	**0.028**
Social Sciences	**IMG vs AMG**	**-24 (-40 to -7.9)**	**0.004**

^a^Male as Ref; AMG as Ref

### Qualitative findings

The qualitative phase of this study explored GP registrars’ perceptions of the relevance, utility, and application of basic science in clinical practice. Of the eleven participants who consented to the interview, six were females, seven were AMGs, and six were recent graduates, within the last five years. Five themes emerged from the qualitative data, including the backbone of clinical medicine, varying utility over time and by specialty, clinical synthesis integrates encapsulated knowledge, professional pressures hinder revisitation of knowledge and knowledge renewal enhances updates.

### Theme 1: the backbone of clinical medicine

The registrars perceived basic sciences as the ‘backbone of clinical medicine’, with the first year of medical training being the ‘gateway’ year that provides the foundational knowledge and the building blocks for the clinical concepts learned in later years. They acknowledged that basic science knowledge played a significant role in clinical reasoning and was applied in clinical diagnoses and decisions.

*I think the main thing that I sort of believe in relation to this topic is that the basic sciences are required before you can have a good clinical understanding, as you would never be able to learn clinical medicine if you didn't have that. You know the backbone information. For example, if you have someone who comes in with acute gout in their toe, you examine the anatomy you explained to them. You know, this is the bone that's affected here. Basically, what happens is you have elevated urine, which forms crystals inside this joint, and that's why it feels like you are walking on glass because you're basically walking on uric acid crystals, glass shards. I think something like that would be an example of where you describe the anatomy of the patient and describe the pathophysiology of what's going on (Participant #1, Male, AMG).*

*For example, some patients come with abdominal pain. If you know the anatomy, then you can try to determine what would be the differential diagnosis according to the location and then you can work it out. So that's one, the other is chest pain, and you know the pathophysiology. So you know it depends on the characteristics of the pain, and you will know whether it is cardiac or from the lungs or whether it is musculoskeletal (Participant #10, Female, IMG).*

Overall, registrars acknowledged that basic science knowledge was applied in clinical practice, often subconsciously. Anatomy and physiology were perceived as the most relevant basic sciences in general practice.

*On a day-to-day basis in any GP practice, I guess it is a theoretical understanding of it [basic sciences] that you apply and draw on that you're not aware of necessarily. (Participant #6, Male, AMG).*

*They all are really, really very important and relevant in general practice. I think the most clinically relevant one is anatomy as I said, you need to know the human body to diagnose the disease source. If you don't know anatomy, you can't examine the patient. You don't know where exactly the pain is (Participant #7, Female IMG).*

*Probably physiology and anatomy together because it's an understanding of those two that gives you a good understanding of how and why different diseases present the way they do, and that also helps you keep communicating those things to the patient as well. The pharmacology and pathology, while there are elements that are important to know. I don't find like I'm drawing those skills quite as much as I would that for the other areas in my usual practice (Participant #4, Male, AMG).*

### Theme 2: varying utility over time and by specialty

The registrars acknowledged that the relevance of basic science knowledge varied over time and across different medical sub-specialities. Some disciplines, such as biochemistry, were seen as less relevant to GPs but crucial for intensive care unit (ICU) or emergency department (ED) physicians. Registrars relied heavily on pathologists’ reports and seldom used their pathology knowledge in practice. Similarly, they used guidelines and resources to inform drug choices, indicating less reliance on their pharmacology knowledge.

*I think [biochemistry] is important, but because it is typically studied at the beginning of university and then studied less and less and it is emphasised less and less throughout university and then into your career, that it does start to fade into the past (Participant #2, Male AMG).*

*I can only speak from a generalist perspective. I'm not a cardiologist or a surgeon. And I'm sure that there will be different areas of the basic sciences that would be useful for different areas of medicine, which therein lies the challenge of what to feature in medical school, you know, to get to cover all bases (Participant #5, Male, AMG).*

*I think I always struggled with biochemistry itself as a subject in medical school. I still cannot remember the Kreb cycle or the exact cycle of the things we covered in biochemistry. I have no idea why it was important to me, but it is important to the ICU physicians because they manage patients based on those little molecular details, whereas it isn’t important to me (Participant #8, Female, IMG).*

*Now I know that I used to use biochemistry a lot more when I was working in emergency full time because you're dealing with derangements in potassium or sodium, whatever it is, or sugar, you know, all the time (Participant #6, Male, AMG).*

### Theme 3: clinical synthesis integrates encapsulated knowledge

Despite some basic science knowledge being perceived as forgotten or less utilised, participants indicated that this knowledge had become integrated and was being used subconsciously in clinical practice.

*I think they’re probably to a certain level and there's this subconscious foundation that's all that other information that you've built along over the course of many years of study. You don't actively think about it all the time anymore because you don't find it quite as relevant to the patient in front of you. They are definitely things that I draw on, but I probably do not actively consciously think back to them (Participant #4, Male, AMG).*

Furthermore, the participants stated that they had learned how to create patterns, filter information and relate basic science information to clinical cases.

*I think I've filtered out all the tiny little details. I think I've also filtered to the point where I only consider things that are important in clinical practice (Participant #3, Female, AMG).*

*That's really tricky because the further you go through a career, you become less reliant on that side of things and are more reliant on what works and patterns. You know, for example, almost all clinicians utilise paracetamol every day. No one knows how it works, but we know it works (Participant #5, Male, AMG).*

### Theme 4: professional pressures hinder revisitation of knowledge

The registrars recognised the relevance and utility of basic sciences in clinical practice, but identified professional pressures, such as lack of time and heavy workload, as barriers to revisiting this knowledge. The vast scope of medical knowledge and lack of readily accessible resources further compounded these challenges.

*It's probably a lack of time for me because there is so much to do, work and life, and then there is so much to learn at a higher level than just finding the time to go back to fundamentals I find hard. So, it would be time based, but also, I don't really know where to look. So, for anatomy, I will just Google image of things so that I can have a look at the anatomy, but I don't really know where I'd go to look for Physiology other than kind of try and go and find PDF's or hard copies of textbooks (Participant #2, Male, AMG).*

*I think one factor would probably be just a short consult length that we have, and the days are pretty busy and sort of, you know, maybe writing down that, oh, I might revisit that, or if you're not sure, I think like I would revisit it personally. But sometimes, if there's something that is not super important for the diagnosis or something, I make a mental note, but then I forget to go back to look it up (Participant #11, Female, AMG).*

*I think it's because, I mean, as a generalist, you need to know a little bit about absolutely everything and be able to draw on more than that little bit of knowledge that you need. When you don't know what's coming, it's very difficult. Whereas when you're a specialist, you know everything about one area, and so you sort of only have to draw on that particular bit of knowledge and a lot of the time in GP practice, you have to do, you don't know what you're going to be dealing with. So you don't have any time to prepare (Participant #6, Male, AMG).*

Another layer of complexity identified by the participants related to dealing with high patient expectations.

*Sometimes, if the patients appear like they have a very high expectation of a GP to know everything; I think it is a bit challenging for the junior GP to revisit their knowledge in front of the patient. I am sure all the GPs without the patient in their consulting room, they can revisit all of their memories, and find out all the exact things by themselves by looking at their guidelines for sure. But sometimes it's a bit challenging doing that in front of the patient (Participant #9, Female, IMG).*

### Theme 5: knowledge renewal enhances updates

The registrars suggested measures to support continuous updating of basic science knowledge. These included structured Continuous Professional Development (CPD) workshops on basic sciences, webinars, and easily accessible online resources. CPD workshops was the most favoured strategy.

*I would say setting up CPD points for GPs is a good way of maintaining it. That's how you get a GP to attend all those teaching and learning activities (Participant #9, Female, IMG).*

*For example, webinars. People are doing a lot of webinars that they can sit at home and then attend the classes and they will get the educational hours (Participant #10, Female, IMG).*

*I mean, I think it would be really handy to have some sort of online resource that is very basic, very user-friendly. To teach, you know, like having pictures of anatomy that you can point out to say, this is what it looks like, or this is the procedure that I'm gonna do, and this is why. Just like very simple application of it all, which has all the useful information like frequently asked questions about the particular issue that you can sort of just go and find information (Participant #6, Male, AMG).*

#### Triangulation of findings

In the quantitative findings, participants scored higher in biochemistry and lower in anatomy, indicating better retention of biochemistry. However, interviewees perceived anatomy and physiology as more relevant to clinical practice. This suggests that perceived relevance doesn’t equate to knowledge retention, possibly due to limited revision opportunities. Biochemistry and pathology, which were considered less relevant, may have been encapsulated and subconsciously integrated with clinical knowledge. This is likely due to regular revisits via readily accessible guidelines and pathologist reports, resulting in better performance and retention of knowledge. Furthermore, the AMGs outperformed the IMGs in most basic science disciplines, indicating higher retention of the basic science knowledge. This could have been because the AMGs, on average were younger with median age of 30 (IQR: 28-34) compared to median age of 36 (IQR: 32-40) for the IMGs and they had lesser years since graduation - median of 4 (IQR: 4-5) versus the IMGs' median of 11 (IQR: 8-16).

## Discussion

This study employed a mixed methods approach to investigate the influence of variables such as age, graduate type, years since graduation, and gender on GP registrars' retention and perceived utility of basic science knowledge in clinical practice. The findings identified gender and high scores in formative clinical examinations as predictors of basic science knowledge retention. Males outperformed females, particularly in anatomy, aligning with existing research that shows males generally excel in spatial tasks within this discipline [24–27]. Interestingly, performance in the formative clinical examination also forecasted competencies in biochemistry, pathology, pharmacology, and social sciences, underscoring the interplay between clinical acumen and basic science proficiency, suggesting a synergistic educational approach [28]. Further research is needed to fully understand these gender disparities and their implications in medical education.

Performance disparities in relation to cultural background were also evident, IMGs scored lower in physiology and social sciences but higher in biochemistry compared to AMGs. This variance could stem from the recency of basic science education among AMGs and the challenges IMGs face due to curriculum differences and adaptation to new medical systems. Most IMGs studied basic sciences early in their education without subsequent reinforcement, contributing to lower retention rates, as highlighted by Custers et al. [29]. Furthermore, the predominantly didactic and teacher-centred approaches in medical schools from the Gulf Countries, Asia, and Africa contrast with learner-centred strategies such as problem-based learning (PBL) which are employed in the UK, USA, Australia, and parts of Europe, to foster better integration of basic sciences with clinical sciences and promote lifelong learning [30, 31]. Thus, enhancing continuous engagement with basic sciences through modern educational techniques is crucial for improving knowledge retention.

While registrars generally excelled in biochemistry despite its perceived low relevance, they struggled with anatomy, which was deemed the most applicable and essential discipline. This paradox may be attributed to the nature of the subjects. Biochemistry, though abstract and challenging, builds on foundational concepts that facilitate new learning and deeper understanding through a constructivist model. This model encourages the integration of new information with existing knowledge frameworks, enhancing retention over time [32–37]. Conversely, the direct application of anatomical knowledge in clinical practice highlights its critical role in understanding pathophysiology and guiding pharmacological interventions, underscoring the integrated nature of clinical reasoning, where biochemical and pathological knowledge are essential, albeit often subconsciously [38, 39].

The learning process for anatomy involves both rote memorisation and deep learning strategies due to its extensive factual content and specialised vocabulary [40–42]. However, studies suggest that initial memorisation strategies do not support long-term retention, as students often rely on assessment-driven motivation rather than genuine understanding, which fails to promote sustained knowledge [43]. The relevance of anatomy is often only fully appreciated after significant clinical experience, indicating a need for educational strategies that encourage ongoing engagement with anatomical knowledge throughout medical training and practice [43]. This calls for re-evaluation of how anatomical knowledge is taught and revisited in clinical settings to bridge the gap between initial learning and practical application.

Despite the variation in perceptions across disciplines, registrars generally acknowledged the fundamental role of basic sciences in clinical reasoning and patient communication. Echoing this result, academic physicians affirmed the value of basic science knowledge in enhancing clinical diagnosis and improving patient interactions [44]. The study further highlighted that the ability to revisit or revise basic sciences in practice is often hindered by factors such as time constraints, workload, and the sheer breadth of medical knowledge. Challenges at the point of care include insufficient time, patient complexity, and information overload, with most inquiries going unresolved and only a small fraction of questions answered [45–47]. This underscores the importance of accessible and applicable resources that support continuous learning and knowledge retention at the point of care [48].

To enhance ongoing engagement with basic sciences, strategies such as development of CPD workshops, webinars, and provision of easily accessible online resources have been proposed. Continuous engagement through clinical practice, continued education, and teaching has been shown to enhance knowledge retention, with CPD programs often serving as the primary source for updating information [49]. Medical educators and training programs are advised to design interactive, multimodal CPD courses that focus on basic sciences to ensure knowledge enhancement and retention [50]. However, considering the busy schedules of registrars, training colleges and programs should integrate clinically relevant basic science courses into required CPDs. Additionally, there is a need for reliable information systems aligned with point-of-care needs that are relevant to practice, aiding clinical decision-making [51–53]. Collaborative efforts between information system designers and GPs could aid the development of resources tailored to specific basic science learning needs, enhancing the practical application of such knowledge in clinical settings [54].

### Strengths and limitations

To the best of our knowledge this is the first study that examined knowledge retention and perceived relevance of basic sciences among GPs. The comprehensive mixed methods approach utilised incorporated quantitative and qualitative data to provide an understanding of basic science knowledge retention and its perceived utility in the GP setting. However, the study is not without limitations. The study assessed a sample of GPs located in one training program and results may not be generalisable to other clinical settings. Therefore, the findings need to be interpreted with caution. Whilst we ensured that the interviews included participants with different characteristics, there might have been bias introduced in the interviews based on recency of graduation with most of the participants graduating within the last five years. This selection may not have accurately representd the broader population of registrars who participated in the quantitative phase of the study.

### Implications for practice and future research

Overall, the findings of the study highlight the need for medical curricula that better integrate basic sciences with clinical practice from undergraduate to postgraduate training years. It is evident that revision of the basic sciences should be continued even after the early undergraduate years as some concepts in the early years may not have been fully reinforced. Medical educators should consider the use of a spiral curriculum which involves iterative revisiting of topics with each encounter building on the previous knowledge [43, 54]. This approach will enhance deep learning, reinforcing prior knowledge and stimulating integration of knowledge [44, 54]. For GPs, the development of accessible, relevant, time-efficient continuing medical education resources could mitigate the barriers to knowledge retention [52]. Future research could explore longitudinal trends in basic science knowledge retention among GPs and investigate the efficacy of different educational interventions in enhancing the application of this knowledge in clinical practice. In addition, studies could also examine the specific challenges faced by IMGs in retaining and applying basic science knowledge, with the aim of developing targeted support mechanisms.

## Conclusion

The findings of the study showed that basic science knowledge retention among GPs varied by discipline. Basic sciences were considered clinically relevant and identified as the backbone of clinical medicine. Nonetheless, time constraint, workload and vastness of medical information were barriers that limited revision of basic sciences in clinical practice. Strategies proposed include development of CPDs and clinically relevant online resources. The findings highlight the need to ensure that basic sciences education is not only retained but effectively integrated. Future research could explore innovative educational strategies that enhance GPs' retention of basic science knowledge in clinical practice.

### Supplementary Information

Supplementary Material 1. Good Reporting of A Mixed Methods Study (GRAMMS) checklist.

Supplementary Material 2. Qualitative Interview Guide.

## Data Availability

The data can be obtained from the corresponding author on reasonable request.

## Abbreviations

CPD: Continuing Professional Development
GPs: General Practitioners
GPT: General Practice Training
MCQ: Multiple-Choice Question
PGY: Post Graduate Year
