# Supplementary material for: The perceived relevance, utility and retention of basic sciences in general practice
Source: BMC Med Educ. 2024 Jul 29;24:809. doi: 10.1186/s12909-024-05750-2 (PMC11285199; doi:10.1186/s12909-024-05750-2)
Supplement: Supplementary file 1 — Supplementary Material 1. Good Reporting of A Mixed Methods Study (GRAMMS) checklist. [file 12909_2024_5750_MOESM1_ESM.pdf]

Supplementary File 1. Good Reporting of A Mixed Methods Study (GRAMMS) checklist

**Guideline**	**Section and Line**
Describe the justification for using a mixed methods approach to the research question	Methods: Lines ~~98 - 110~~
Describe the design in terms of the purpose, priority and sequence of methods	Methods: Lines ~~103 - 110~~
Describe each method in terms of sampling, data collection and analysis	Methods: Lines ~~132 - 190~~
Describe where integration has occurred, how it has occurred and who has participated in it	Methods: Lines ~~188 - 190~~<br>Results: Lines ~~415 - 452~~
Describe any limitation of one method associated with the present of the other method	Discussion: Lines ~~585 - 591~~
Describe any insights gained from mixing or integrating methods	Discussion: Lines ~~434 - 579~~

Reference: O'Cathain A, Murphy E, Nicholl J. The quality of mixed methods studies in health services research. J Health Serv Res Policy. 2008;13: 92-98.
