# Supplementary material for: The perceived relevance, utility and retention of basic sciences in general practice
Source: BMC Med Educ. 2024 Jul 29;24:809. doi: 10.1186/s12909-024-05750-2 (PMC11285199; doi:10.1186/s12909-024-05750-2)
Supplement: Supplementary file 2 — Supplementary Material 2. Qualitative Interview Guide. [file 12909_2024_5750_MOESM2_ESM.pdf]

Supplementary File 2. Qualitative Interview Guide

1. Can you tell us more about how you engaged with basic science as a student?
2. How did you engage with it as a doctor? Have you had the opportunity to revisit the knowledge from medical school in your clinical practice?
3. Please recall the questions you had in the basic science exam, what was your perception of their clinical relevance?
    a. Which of these would you have typically encountered in practice?
4. In your experience, have you found a particular basic science discipline to be more applicable or useful in your clinical practice?
    a. Does it have any impact on your clinical decision-making and patient care?
    b. Please could you provide some examples of how knowledge of these basic science disciplines (knowledge of anatomy, biochemistry, physiology, pharmacology, and/or pathology) has helped you make important clinical decisions and was crucial for successful patient care?
5. What are the challenges or barriers that prevent GPs from upskilling or revisiting basic science knowledge?
6. How do you see the integration of basic science knowledge into clinical practice?
    a. How can clinicians make the most of this knowledge to improve patient care?
7. What strategies do you think can be implemented to ensure that practitioners maintain an up-to-date knowledge of these basic sciences throughout their careers?
8. Is there any other comment you would like to add about the relevance of the basic sciences in relation to clinical practice?
